# Abstracts and Gleanings

**Published:** 1880-03-20

**Authors:** 


### ABSTRACTS AND GLEANINGS.

**Nasal Catarrh.**—In a discussion on a paper read by Dr. Sterling in the society of the county of Kings, Brooklyn, Dr. S. Sherwell said he did not believe that naso-pharyngeal catarrh had ever caused a case of tuberculosis—at least he had never seen one. He agreed with the reader, however, that it will help to produce phthisis; but not that naso-pharyngeal catarrh is an essential factor in its production, or that it leads directly to phthisis. He did not think it is so claimed by any laryngologist, and certainly they ought to know something about it.

Dr. J. H. H. Burge referred to the statement that a vast majority of cases of this class are in the hands of quacks. It reminded him that one of the most active ingredients in one of the most popular nostrums for the cure of this disease is salicylic acid. He had no doubt but that its influence on the disease was good. He had tried it himself with benefit.

Dr. Sherwell was of the opinion that the chief nostrum of this character contained principally nitrate of silver.

Dr. Burge thought not—at least the nostrum to which he referred contained none. He could readily distinguish between salicylic acid and nitrate of silver. The skin about the nose was not turned black by the action of light.

Dr. W. H. Bennett fully agreed with the author of this paper as regards the influence of dust in the production of naso-pharyngeal catarrh and phthisis. He referred not to tuberculosis, but to catarrhal and fibroid phthisis. In the year 1872 he called the attention of the profession to the influence of street dust in the production of disease. In making laryngoscopical examinations he had noticed dust on the surface of ulcers in the larynx and trachea forty-eight hours after exposure. He had noticed the same circumstance in regard to the vomer, when making rhinoscopic examinations. There can be no doubt of the fact that dust caused catarrhal and fibroid phthisis. Catarrh affects the lungs in those persons suffering therefrom who breathe the air which passes over putrescent nasal secretions, and they must necessarily experience the evil result. The speaker had experimented on the subject of dust as a causation of disease. He had confined animals in a room in which particles of dust were caused to float in the air, and none lived beyond twelve months. It seemed to him that catarrh might be divided into different varieties. 1st. That variety in which there is an abundance of secretion. 2d. A variety in which there is scarcely any secretion; there appears to be simply a cell proliferation; and 3d. A variety in which the glandular apparatus is more or less involved. Almost all cases of chronic catarrh become, sooner or later, cases of proliferous disease, the secretion becomes diminished, and then hypertrophy of the infiltrated cells appears. In regard to the treatment, he had only to say that, in his estimation, it is a subject which demands more attention than it ordinarily receives.

Dr. A. Mathewson: The treatment of the air passages consists largely in the treatment of the pharynx. He did not accomplish very much if he did not take this fact into consideration. Fully 75 per cent.

of catarrhal cases involve the pharynx. As a practical point, in the application of fluid remedies to these parts, it is well to direct the patient not to blow the nose, or cough, for a short time after the application. Cases are quite common in which severe irritation or inflammation of the middle ear results, the fluid being forced up through the eustachian tube by the expulsive efforts above referred to. Even snuffing so simple a mixture as salt and water is sometimes quite as bad as blowing the nose. He is always very careful, after applying a fluid, to direct the patient to wait some time before blowing the nose.

Dr. E. R. Squibb referred to experiments now being made in France with dust, the object being chiefly to discover bacteria. So far as he knew, bacteria are not found in nasal and buccal secretions. A nice way to filter the air is to fasten a piece of dampened sponge before the nostrils. A person using this apparatus (which is largely used in pharmaceutical manufactories, while working in substances of a more or less dangerous nature, such as aconite root, and the like) will find an abundant collection of dust on the sponge, for the reticulation of the sponge is very great, and will prevent dust from entering the air passages about as effectually as almost any substance.

Dr. J. Walker spoke of overheated houses as a prolific source of catarrh. Persons thus overheated will incautiously expose themselves to a colder temperature. A very common cause of catarrh is the dust which arises from and through old, dusty, rusty furnace flues. Rusty furnaces, becoming so through the summer, are especially bad in this regard. Then, again, the air chambers of furnaces do not, in all cases, communicate with the open air. They open into the cellar, and take up the unhealthy air of cellars, with all its odors and poisons, as, for instance, from decayed vegetation and the like. He had in his mind a case where the air chamber of a furnace was two or three feet from the wall. The speaker knew of a row of houses built upon the same plan—which is a dangerous one, and ought to be remedied.

The Chair said Dr. T. R. French refers, in a note, to the use of the Thudicum douche in the treatment of catarrh. He says: "I have used the douche in the treatment of chronic nasal catarrh and eczema in 165 cases, and have yet to see the first case of middle ear trouble, such as Dr. Roosa and other ear men tell us is apt to follow the use of this instrument. This I attribute largely to the care taken in directing the patient in its use. If care is not taken by the physician in directing, and by the patient in using the douche, I can understand how injury might be done to the ear. I use the Thudicum douche in my hospital clinic only; for, when my patients can afford it, I am in the habit of employing a spray douche, which has many advantages over the Thudicum douche." He asked Dr. Mathewson to state whether there was any greater danger in the use of this than other moist applications.

Dr. Mathewson thought there was. He had reported several cases in connection with Dr. Roosa, where serious middle ear troubles had resulted from the use of the douche. One or two of these cases were physicians, who would be supposed to exercise great care in its use. One physician, who had taken part in a discussion of this subject, used the douche afterwards, but troubles of the middle ear was the consequence. So many cases have occurred in his practice, that his convictions are stronger and stronger that there is danger in the use of fluid applications if the patient blows the nose too soon after the operation. This is in consequence of the peculiar anatomical arrangement of the nasal air passages. The spray is not so apt to produce bad results. It would not be likely to send a current into the middle ear; or if it did, fluids in the form of spray are not so dangerous as otherwise.—*Proc. of Med. Soc. of the county of Kings.*

**Recent Decisions.**—The following are notes of decisions by the courts concerning matters of medico-legal interest:

1. In the trial of a man indicted for rape, the complainant testified that the defendant and some others seized her on the street at night and carried her into an alley-way, where he and the others ravished her. There was a verdict of guilty, and the defendant moved for a new trial. The defendant requested the court to charge the jury that, to constitute the crime of rape, it was necessary that the prosecutrix should have manifested the utmost reluctance, and should have made the utmost resistance. The court did not comply with this request, and the refusal to do so was made the ground for asking a new trial. The importance of resistance was held by the supreme court of appeals, before which the motion came, to show two elements in the crime: carnal knowledge by force by one of the parties and non-consent thereto by the other; and the jury must be satisfied of the existence of these two elements in every case, by the resistance of the complainant if she had the use of her faculties and physical powers at the time, and was not prevented by terror or the exhibition of brutal force. So far, resistance by the complainant is important and necessary; but to make the crime hinge on the uttermost exertion the woman were capable of making would be a reproach to the law as well as to common sense. Such a test it would be exceedingly difficult, if not impossible, to apply in a given case. If the failure to make extreme resistance was intentional, in order that the assailant might accomplish his purpose, it would show consent; but without such intent it shows nothing important whatever. A new trial was not granted.

2. A man murdered a woman by shooting her; his defense was that he was intoxicated, and thus irresponsible. The case came before the Nebraska supreme court on exceptions filed by the defendant. The court reaffirmed the principle, now tolerably well established, that "settled insanity, produced by intoxication, affects the responsibility in the same way as insanity produced by any other cause; but insanity immediately produced by intoxication does not destroy responsibility when the patient, when sane and responsible, made himself voluntarily intoxicated."

In the same case, it was held that the fact that the prisoner was in a drunken state when he committed the homicide does not itself render the act of shooting the deceased any the less criminal, nor is it available as an excuse.

3. A midwife volunteered to cure an attack of ophthalmia in the infant at whose birth she assisted, and whom she was nursing. She advised the parents that it was unnecessary to call in a physician, as she had successfully treated similar cases. The sight of the child was destroyed. The mother of the child brought an action to recover damages. At the trial there was medical evidence that if other and more active remedies had been used loss of sight would not have resulted. The midwife did not pretend to know of these remedies.

The case was carried from the superior to the supreme court on exceptions, the lower tribunal having dismissed the suit with a verdict for the midwife, without giving the case to the jury. The supreme court confirmed this disposition of the action, on the ground that a person who without special qualifications volunteers to attend the sick can at the most be required to exercise the skill and diligence usually bestowed by persons of like qualifications under like circumstances. Under the rule requiring ordinary care, as applied to this case, the court saw no evidence of neglect in any degree. [That is to say, the midwife showed no greater skill than she pretended to possess,—a safe harbor for incompetency.]

4. The liability of hospitals for the consequences of the acts of their visiting physicians and surgeons on duty has recently been tested anew in a case in New York, and the principle is reaffirmed that if such institutions have exercised due diligence in securing skillful and careful medical men to treat their patients, they cannot be held accountable for alleged malpractice on the part of those medical officers.

One of the surgeons of the Manhattan Eye and Ear hospital, after consultation with both his colleagues, advised and performed an operation for chronic glaucoma. The necessity of an operation was agreed to by the three consultants, and there was no accident during its progress. Two weeks later the patient left the hospital with vision exactly the same as when she entered. In a few days after her discharge, inflammation developed, and she returned to the hospital for treatment. Another operation was advised, and the patient consented to its partial performance. The operation had no permanent beneficial effect, and the woman became blind. She brought a suit against the hospital for fifty thousand dollars for the loss of her sight. The trial took place before the New York supreme court, Judge Lawrence presiding. After a full hearing of the testimony, in which, on the part of the hospital, it was shown among other things that no complaint was made at the time of the alleged injury to the surgeons or to any of the hospital authorities concerning the surgical treatment; that the surgeons were men of preeminent skill in their profession, and had pursued the usual methods of diagnosticating and treating chronic glaucoma— a disease which almost invariably ends in blindness—the judge dismissed the complaint without giving the case to the jury. This decision in favor of the hospital rested on and reaffirmed the principles set forth in a judgment rendered by the supreme court of Massachusetts in a similar case, the essential points being that the Manhattan Eye and Ear Hospital was a charitable institution which, having exercised due care in the selection of its agents, was not liable for any injuries to a patient caused by their negligence; that, in the present instance, there was no proof that there had been any negligence whatever on the part of the surgeons, who were shown to be men of superior skill, and to have exercised their skill with proper caution.—*Boston Med. and Sur. Jonrnal.*

**Salicin and Salicylic Acid in Acute Rheumatism.**—It is a fact that salicylic acid and salicylate of soda not unfrequently give rise to considerable and even alarming depression. Such an untoward effect is not produced by salicin. From a therapeutic point of view this is one of the most important points of difference between the two remedies. In a disease, such as acute rheumatism, in which the heart is apt to be involved, the absence of this tendency to cause depression points out salicin as a much safer remedy than salicylic acid. Its superiority in this respect is specially referred to by Senator, who, curiously, does not seem to see that the fact to which he directs attention is a strong argument against his view that salicin owes its therapeutic virtues to its being converted into salicylic acid in the system.

Of the depressing action of salicylic acid many instances are recorded. Several have come under my own notice. The following is of value as the unbiased evidence of an intelligent, well informed medical man, founded on his own experience of the two drugs. My friend and then neighbor, Dr. Sinclair, of Dundee, now physician to the infirmary of that town, suffered from an attack of subacute rheumatism last December. Before I saw him he had been taking salicylate of soda in twenty-grain doses with relief to the pain; but it so depressed him, and made him feel so wretched, that he said he could not go on with it. I recommended salicin instead. He took it in even larger doses than the salicylate, with speedy relief to his rheumatism and without any untoward effect. On the contrary, he seemed, under its influence, to regain strength and appetite, and was soon quite well.

The following is his own statement, given with his permission :

"Both drugs relieved the pain, tenderness and swelling, when taken in full doses frequently repeated. But the salicylate, which I employed first, produced some very unpelasant effects. The taste I found to be disagreeably sweet and nauseous. After taking several twenty-grain doses, a copious perspiration was produced; the strength of the pulse was very distinctly diminished, while its frequency was increased; and a feeling of most uncomfortable depression, with singing in the ears, ensued. Indeed, I hardly knew whether the disease or the remedy was preferable. Salicin, on the other hand, has a pleasantly bitter taste; it improved the tone of my pulse and digestion, and relieved the pains more rapidly. Neither drug gave any relief except when taken in twenty or thirty-grain doses every hour for from six to twelve consecutive hours. It may be said that, had I taken smaller or less frequently repeated doses of the salicylate, I might have escaped all the disagreeable effects except the taste—itself no small matter. But such doses produced no effect on my rheumatism. To my mind one of the great merits of salicin is the absolute safety with which large doses can be taken. In the course of one period of twenty-four hours I swallowed an ounce of it with nothing but benefit."

I have seen salicylate of soda produce very alarming depression, closely resembling that of the typhoid state. Not long ago I saw in consultation a case in which it was a question whether the fatal result was not due to the depressing action of the salicylate. By some this effect has been attributed to the presence of carbolic acid, consequent on faulty preparation. Such an explanation may have been applicable to some cases, but is not so to all. I have more than once seen marked depression produced by a solution of salicylate of soda in which no trace of such impurity could be found, and which was given to another patient without causing any unpleasant effect. The worst effects that I have ever seen follow the administration of large doses of salicin are a sense of fullness in the head and singing in the ears; such symptoms are commonly produced by large doses of quinine.—*London Lancet.*

**Tracheotomy in Croup.**—Steiner, a recent contributor, writes "as to the time when tracheotomy is to be performed, I agree with those who urge an early operation, and do not defer it until urgent symptoms of carbonic acid poisoning have manifested themselves." "The beginning of the third stage—when remissions in the paroxysms of dispnœa begin to grow less frequent—is the proper one for the operation." Hardy and Beheir say, operate when the symptoms indicate extension of the false membrane. Gross, Ollivier, Trousseau, Bryant, Neimeyer, Roberts, Aitken, Agnew, Packard, all advocate early operation. Pilcher expresses himself, "justice to my patient, justice to myself, fidelity to the profession I represent, all unite in demanding that now, early, before the development of conditions which will make any interference but a forlorn hope, tracheotomy should be done." Age for operating.—Tables show between 7 and 8 years as the best period, then comes 6 to 7 years, then again, between 3 and 5 years, operations under 2 years promising the fewest chances. In a table of 32 operations under 2 years there were 5 cures and 27 deaths.

*Remarks.*—It has been shown that this collection embodies eight hundred and sixty-three operations, with six hundred and eighty-five deaths and one hundred and seventy-eight recoveries, making the proportion of cures as one to every four and three-fourths cases; but the proportion of deaths is unnecessarily increased by forty cases which should be excluded, since death in them was attended by such complications that their exclusion from the list seems warrantable. The complications of which I speak are, namely: Death by anæsthetics 3; moribund at operation 21; death from scarlatinal poison 4, from choking of a home-made tube 1. from carelessness of the nurse—letting canula become displaced—3, from tube getting plugged through carelessness of the nurse 2, from erysipelas 1, from outside complication 4, and from convulsions due to indigestible food 1.

This makes the proportion as one hundred and seventy-eight cures to eight hundred and twenty-three operations (one in a little over four), instead of one hundred and seventy-eight recoveries to eight hundred and sixty-three tracheotomies; which I regard as the correct average of success.

Again, an estimation of the recoveries, in proportion to the operations performed, between the Northern and Southern States was made, but only negative results were obtained on account of the insufficient data—the proportion of cures being nearly equal in the two sections.

From this somewhat hurried analysis of these statistics, with the generally received opinions on the subject of tracheotomy, the annexed conclusions are deducible.

*First.*—That tracheotomy is *per se* almost devoid of danger;

*Second.*—That fatal hemorrhage should almost never occur; and care with coolness will nearly always prevent apnœa from intra-tracheal bleeding;

*Third.*—That age offers no contra-indications, although the average of success is less in early infancy and adult life;

*Fourth.*—That early operative interference—whenever the paroxysms of dyspnœa become at all lengthened—is demanded, since delay only adds to the suffering of the patient, and materially lessens the chances of recovery; and

*Fifth.*—That the after attention is of prime importance; careful attention to the wound, proper treatment of the disease, and proper nursing with fair hygienic surroundings, being the essentials to a successful issue.—*Gaillard's Med. Journal.*

**Irritative Cough from Elongated Uvula—Operation.**—This child, about seven years of age, presents an illustration of a persistent dry cough without any pulmonary disorder. The explanation, however, is at once apparent upon directing our attention to the throat. An elongated uvula rests on the base of the tongue, and constantly titillates the entrance of his larynx, thus setting up spasmodic cough. From debility of the parts, which is generally accompanied by more or less inflammation or chronic sore throat, the soft palate becomes relaxed, and the uvula drops down upon the tongue; or the uvula itself may be hypertrophied and elongated, as in the present case. This condition is most likely to be set up in young subjects, although it may occur at any time of life, and is often found associated with a strumous diathesis and a delicate constitution.

This apparently trifling affection may produce considerable inconvenience. Hawking, coughing, constant irritation, sense of strangulation during sleep, and nightmare, are among the immediate results; among the later ones may be feared the occurrence of tubercular deposits in the lungs.

If the disorder be due simply to a relaxation of the soft palate, which often occurs in consumptives and dyspeptics, the use of astringent gargles and applications—among the best of which may be named nitrate of silver solution, gr. xx to gr. xxx, applied with a swab twice a week—may be followed by relief from the symptoms. But when there is a marked hypertrophic elongation of the uvula, the proper remedy is the removal of a considerable portion of the organ, which is readily accomplished with the scissors. For this little operation no chloroform will be needed, except where the patient refuses to co-operate with the surgeon. Sometimes in young children much trouble is experienced from their active struggling, and then the operation is greatly facilitated by a little of the anæsthetic.

I have now performed the operation upon this young lad, and would have accomplished it more satisfactorily if he had not resisted me. No hæmorrhage will occur after gargling with a little cold water or vinegar and water. I shall insist upon the importance after this operation of his using a liquid diet for a few days, and of being careful not to catch cold.

*Remarks upon Chloroform.*—In regard to the administration of the anæsthetic, you should not forget that chloroform should never be given with the patient in an erect, nor even in a semi-recumbent posture. Owing to the tendency to syncope and heart-failure, the head should not even be raised from the pillow, nor the neck bent. Of course you would not give chloroform nor any anæsthetic immediately after a full meal, on account of the danger of incomplete vomiting and strangulation. No food should be given for at least four hours before the administration of chloroform. The assistant in charge of the anæsthetic should devote his entire attention to watching its effects upon the patient, and should not look at what the surgeon is doing. The administration must not be hurried; chloroform must not be crowded, but given deliberately and with plenty of atmospheric air. In regard to the amount necessary to be used, in the case of an infant, you have noticed that only a few drops are placed upon the centre of a folded towel, in the manner in which you have frequently seen it done by my experienced assistant, Dr. Hearn; for an adult the amount may be increased to half a drachm at first, to which a few drops are added from time to time to supply the loss by evaporation. The clothing must be loose about the chest and the abdomen during the administration. Should a change be noticed in the pulse or appearance of the patient, the chloroform must be at once removed and the patient turned upon his side, the tongue drawn forward and the face dashed with cold water; and the chest, or, in the case of a child, the nates, well wiped with a fringed towel wet with ice-water. If the patient does not revive, the foot of the table may be elevated so as to allow the head to hang down, or the patient may be lifted by the heels, or "inverted," while artificial respiration is attempted. The vapor of nitrate of amyl, or spirits of ammonia, may be cautiously given, which sometimes has a remarkable effect.

With care in administration a fatal result may generally be averted, especially if the tendency to syncope be borne in mind, and prompt measures taken to overcome it. By pursuing the methods just laid down, I have successfully administered chloroform in probably more than five thousand cases without a single fatal result. Chloroform should be administered with especial care to haoitual drinkers, and to those who are the subjects of heart or kindey disease. It seems to be particularly applicable to young and middle-aged persons. In strong adults, it occasionally happens that we cannot make them unconscious with ether, and we are obliged to give them a small amount of chloroform in addition.

Although chloroform does not commonly cause vomiting, and is much more pleasant and efficient than ether, I do not now use it as frequently as formerly, but have yielded my preference in deference to popular opinion, which at present holds the surgeon responsible if any accident should happen. I therefore employ the safer but less agreeable agent to a very great extent, as a substitute for chloroform.—*Prof. Gross in Jeff. Med. Coll.*

**Forgetting Personal Identity.**—Observation has demonstrated that portions of the brain may be injured or paralyzed, and some quality or function of the mind affected, while in other respects the person may be perfectly sound. Phrenologists explain this by the hypothesis that those portions indicated by certain "bumps" if injured would correspondingly control the acts of the individual. I am inclined to believe that this theory contains a good deal of truth.

An example came recently under my notice illustrating a derangement of mind in one particular, whereas in other respects the condition of the person was perfectly sound, mentally and physically. He is 35 years old, a watchmaker by trade, and an old resident of Cleveland. He has always enjoyed good health, is bright and active intellectually, an original mechanical genius in his way, of strict business integrity—in short, one of our best citizens. Almost a year ago he suddenly disappeared. Search proved unavailing. The river and harbor were dragged, but no trace or tidings ensued. His business was in excellent condition ; domestic matters were unusually agreeable. No cause could be assigned for his extraordinary absence, except violence or self-destruction.

Six months had elapsed when his family first obtained a clue to his movements. He had a child, a daughter six years of age, whom he loved with a most ardent affection. A note was received by her, brief, without date or other index to show his identity; even the post-mark was a blotch of ink that precluded every hope of ascertaining the place of mailing. The missive simply "congratulated her upon her approaching birthday, and regretted that he had no presents to send her." There was no signature, though the address to her name, number and street, city, county and state, was properly made and without inaccuracy.

Hope was revived that he was alive somewhere. By means of extensive advertising, the missing man was at length discovered in one of the western counties of Ohio, an inmate of a public institution. He had come thither, having left Cleveland by a night train and riding ninety miles. Leaving the cars he repaired to the nearest hotel. In reply to inquiries, he stated that he had lost the knowledge of his own identity ; he could not tell his name, or whence he came, nor how he came thither ; that he had no recollections as to where he lived, nor of his family, if he had any. He seemed to be in a lost state of mind and full of anxiety—all appeared strange to him. From his gentlemanly appearance the landlord kindly kept him a few days. One evening he attended a temperance lecture and became somewhat excited—he rushed forth into a neighboring saloon and smashed in the windows, and tore around generally, was arrested, and the court sentenced him to the county infirmary. On political questions of the day, or business matters generally, he would converse rationally and intelligently, no one suspecting the least trouble with his faculties. While in the infirmary he would dismantle a watch or clock, inspect its parts minutely, clean them and put them all together again with the utmost nicety and precision. But for the entire year that he was there he never uttered his own name, or gave any idea of his residence or identity.

When at last a most intimate friend called to take him home to his family and friends he seemed strangely puzzled. He could not recognize the name ; said he had never before heard it. When his wife's name was mentioned he could not think who it was, and said he had no knowledge of anything that had passed, and did not know he came there.

Upon arriving at home he recognized no one, and could not mention the name of a single individual. He would go about the streets and into stores and frequented places with as much regularity and system as ever. But his skill in *dissecting* a watch, and repairing, seems to be heightened, and his sense of touch and accuracy in adjusting its parts seems to have acquired a most wonderful and miraculous perfection. Can any one of my scientific brothers of the profession inform me satisfactorily what portion of the brain must be diseased to produce such a state of mind as I have above related?—*Dr. A. G. Springsteen in Med. Tribune.*

**Intra-Uterine Medication.**—Dr. Lombe Atthill, in British Medical Journal, says, that when dealing with cases exhibiting symptoms of intra-uterine diseases, he invariably directs his treatment to the diseased surface. The symptoms are :

1. Derangements of the menstrual functions.
2. Uterine catarrh.
3. Pain, specially if caused by the passage of the uterine sound.

Fluids should never be introduced into the cavity of the uterus unless the cervix be first freely dilated ; the treatment is in any case unsafe and objectionable. Ointments are difficult of application and inefficient ; so are powders.

Dr. Atthill uses the following agents only : carbolic acid, tincture of iodine, iodized phenol, nitric acid solid nitrate of silver, zinc points, crayons of iodoform. In using carbolic acid, the probe by means of which the agent is carried to the fundus must be passed up twice, for much of the acid with which the cotton is charged is neutralized as it passes through the cervical canal the first time. The second application gives more pain than the first, but this soon dies away, as the acid is a local anæsthetic.

The preparation used is composed of two parts carbolic acid and one part spirits or glycerine. It should usually be applied every three or four days for some weeks. Iodine is dirty and mal-odorous, but sometimes useful as an alternative. Dr. Atthill uses iodized phenol (iodine one part, crystallized carbolic acid two parts ; mix and combine with gentle heat). Diluted, it is an efficient escharotic, and useful in chronic endomyelitis of old women, in whom a fetid discharge frequently occurs. Nitric acid should only be employed in severe cases. It is very efficient. The patient should be kept quiet a day or longer after the application. It should never be applied except at the patient's house. A canula should be used when it is applied to the interior of the uterus, to protect the cervix, which may otherwise contract subsequently. Solid nitrate of silver and zinc points are useful, but some times cause cellulitis. They are most useful when menorrhagia occurs, the os and cervical canal being patulous, but the uterus not much enlarged, and when the hemorrhage is thought to be due to a vascular condition of the intra-uterine mucous membrane, rather than to the existence of a thickened and granular condition of its surface. They should not be used if copious uterine catarrh be present. Iodoform sometimes allays pain, but is uncertain in its action, and has little, if any, effect as a caustic.—*Med. Times.*

**Hiccough.**—In order to relieve hiccough, inflate the lungs as fully as possible, and thus press firmly and steadily upon the agitated diaphragm. In a few seconds the spasmodic action of that muscle will cease.—*Chicago Med. Journal and Exam.*

**New Treatment of Placenta Previa by Ferri Persulphatis.**—Dr. R. J. Nunn, of Savannah, Ga., reports a successful case in the American Journal of Obstetrics. He used it as follows: I found the pains had entirely ceased, the vagina was filled with clots, the os dilated sufficiently to admit the finger, by which the placenta could be easily detected, and the warm blood could be distinclty felt flowing through the os. Cleaning out the clots, a speculum was introduced, and the liquor ferri persulphatis was applied to the bleeding surface by means of a cotton swab passed through the os. The hemorrhage ceased instantly and absolutely, and the speculum was retained in place about fifteen minutes to see that bleeding did not recur. Stimulants and ergot were then given freely, and a pledget of cotton saturated with the styptic was left in the os, and sustained in place by a very slight tampon of cotton, merely sufficient for that purpose. The liquor amnii had been very slowly discharging for a couple of days. Labor recommenced in about an hour. Up to 6 a. m. no blood was lost, but at this time, during an effort to rise, the tampon dropped out, and with it about an ounce of fresh blood, but no clots. A speculum examination showed the os dilated about one-half, the placenta covering the orifice was now plainly visible, and the blood was flowing from the left margin. The iron solution was again applied, which stopped the bleeding instanter, and hence it was thought unnecessary to use the pledget. At 7:15 the hemorrhage recommenced, but was instantly controlled as before. All this time labor was going on satisfactorilly. At 8:20 the patient got out of bed to have an evacuation, when, during a severe pain, the placenta was expelled, followed shortly after by the fœtus, which was dead, and apparently had been for several hours. The subsequent history of the case has in it nothing worthy of note.—*Toledo Med. Journal.*

**Pulsatilla for Dysmenorrhea.**—Pulsatilla is rapidly growing in favor with many practitioners. Though a very old remedy, having been known to Dioscorides and Pliny, it fell into disuse, if not into disrepute, and was not reinstated till about the beginning of the present century. I have used pulsatilla mainly in simple dysmenorrhœa, and here it has doubtless proved of decided utility.

The tincture of pulsatilla should be made from the fresh plant, and given with caution. The dose is from three to ten drops.—*Toledo Med. Journal.*

**Remedies for Hypodermic Use.**—Recent clinical reports show that the equivalent of fifteen grains of hydrobromate of quinia can be used for hypodermic injection without producing local irritation or abscesses. Baltimore physicians are using this remedy extensively by the hypodermic method.

The orthopnœa of asthma will be relieved in a surprisingly short time by the hypodermic injection of one-tenth of a grain of apomorphia.—*Journal of Materia Medica.*

**Failure of the Audiphone.**—Tests of the audiphone as applied by Messrs. Sharp and Smith, of Chicago, have failed in every one of 150 successive cases of deafness. The use of the instrument was attended by no benefit whatever.—*Journal of Mat. Med.*

**Milk and Lime-Water.**—Milk and lime-water are now frequently prescribed by physicians in cases of dyspepsia and weakness of the stomach, and in some cases are said to prove very beneficial. Many persons who think good bread and milk a great luxury, frequently hesitate to eat it for the reason that the milk will not digest readily: sourness of stomach will often follow. But experience proves that lime-water and milk are not only food and medicine at an early period or life, but also at a later, when, as in the case of infants, the functions of digestion and assimilation are feeble and easily perverted. A stomach taxed by gluttony, irritated by improper food, inflamed by alcohol, enfeebled by disease, or otherwise unfitted for its duties—as is shown by the various symptoms attendant upon indigestion, dyspepsia, diarrhœa, dysentery and fever—will resume its work, and do it energetically, on an exclusive diet of bread and milk and lime-water. A goblet of cow's milk may have four tablespoonfuls of lime-water added to it with good effect. The way to make lime-water is simply to procure a few lumps of unslacked lime, put the lime in a stone jar, add water until the lime is slacked and of about the consistence of thin cream; the lime settles, leaving the pure and clean lime-water on the top.—*Journal of Mat. Med.*

**Poisons and Antidotes.**—In an interesting article on this subject, by Dr. F. A. Falk, referred to in *Schmidt's Fahrbucher*, the writer points out that the so-called antagonism of opium and belladonna was noticed by Albinus and others as early as 1570. A real antagonism does not exist, according to Falk. His conclusions are as follows:

1. Atropin is a true antidote for muscarin, but not the latter for the former.
2. Duboisin is also a true antidote for muscarin.
3. Atropin and duboisin are also antagonistic to pilocarpin.

Atropin and physostigmin,
Strychnine and chloral hydrate,
Morphine and atropin,
are all respectively antidotal in a pharmacological sense, but not in a physiological one; that is, the one will diminish the symptoms caused by the other, but will not produce contrary physiological effects.—*Med. Times.*

**Operation for Pterygium.**—Medical Press and Circular: Dr. Yreau Munar, from Palma, Majorca, describes the following method, which has been employed by him with signal success during six years: Firstly, he detaches the pterygium from summit to base; secondly, he folds it back in such a manner that the point touches the middle of the posterior surface of the base, fixing it in this position by means of two or three sutures. The external surface of the pterygium is thus turned toward the eye.

**Eserine.**—Eserine is one of the alkaloids of the calabar bean. It is most commonly used in the form of a neutral salt—the sulphate of eserine.

It is a myotic, and its local effects are antagonistic to atropine and duboisine.

**Hyoscyamia in Insanity.**—Among the papers of practical interest, in New York Medical Society, was one by Dr. John P. Gray, superintendent of the New York State Lunatic Asylum, on the use of hyoscyamia in insanity.

Dr. Gray gave briefly the results of the experience at the above institution in the use of the drug. He stated that it had been the practice at the asylum, from time to time, to make a study of special remedies "to determine, as far as possible, their therapeutic value and their application to the conditions of the insane."

Of the action of hyoscyamia he said :

"In cases of acute mania and melancholia with frenzy, no remedy we have used has so efficiently and readily calmed the high nervous and muscular excitement, and brought about a degree of tranquillity essen tial to acquiescence in nourishment and rest, as a means of restoration."

It was also found of great value in controlling the cerebral excitement of certain cases where there was persistent refusal of food, as it made it "reasonably easy and entirely safe to introduce the stomach-tube and administer the necessary food."

**Hot Water for the Induction of Premature Labor.**—Dr. Benicker related at the Berlin Obstetrical Society a case of dropsy of the amnios, showing the advantage of inducing premature labor by irrigating the vagina with water at a temperature of 40° R. (122 Fahr.), to which some carbolic acid had been added. Two injections in the evening brought on pains, which increased during the night, and after two other injections in the morning the cervix became well dilated. Dr. Benicker recommends this procedure as an energetic means of exciting labor without injury to mother or child. Its effects will vary according to the degree of excitability of the uterine fibres in different women.

Dr. Runge, who had already published a successful case, cited another, in which the injection failed. All trials that have been made show the harmlessness of the procedure for the child.—*Med. and Sur. Reporter.*

**Aspiration of the Bladder for Retention of Urine.**—Eight or ten cases of retention of urine due to organic stricture of the urethra, with spasm superadded, in which endeavors to relieve in the ordinary way had failed, have been successfully treated by Mr. C. B. Pasley, by aspiration of the bladder immediately above the pubes. The advantages of this plan of treatment briefly are, he says, in British Medical Journal :

1. The operation is very simple, and, if skillfully performed, absolutely without risk.
2. The pain caused by the introduction of the trocar is trifling and momentary.
3. The patient is afforded instantaneous relief.
4. In every case the patient passed urine freely in a few hours.
5. The delay under the usual method of treatment is avoided, and the patient is saved much unnecessary suffering.

